# Supplementary material for: Tankyrase disrupts metabolic homeostasis and promotes tumorigenesis by inhibiting LKB1-AMPK signalling
Source: Nat Commun. 2019 Sep 25;10:4363. doi: 10.1038/s41467-019-12377-1 (PMC6761205; doi:10.1038/s41467-019-12377-1)
Supplement: Supplementary file 1 — Supplementary Information [file 41467_2019_12377_MOESM1_ESM.pdf]

Supplementary information

Tankyrase disrupts metabolic homeostasis and promotes tumorigenesis by inhibiting LKB1-AMPK signaling

Li et al.

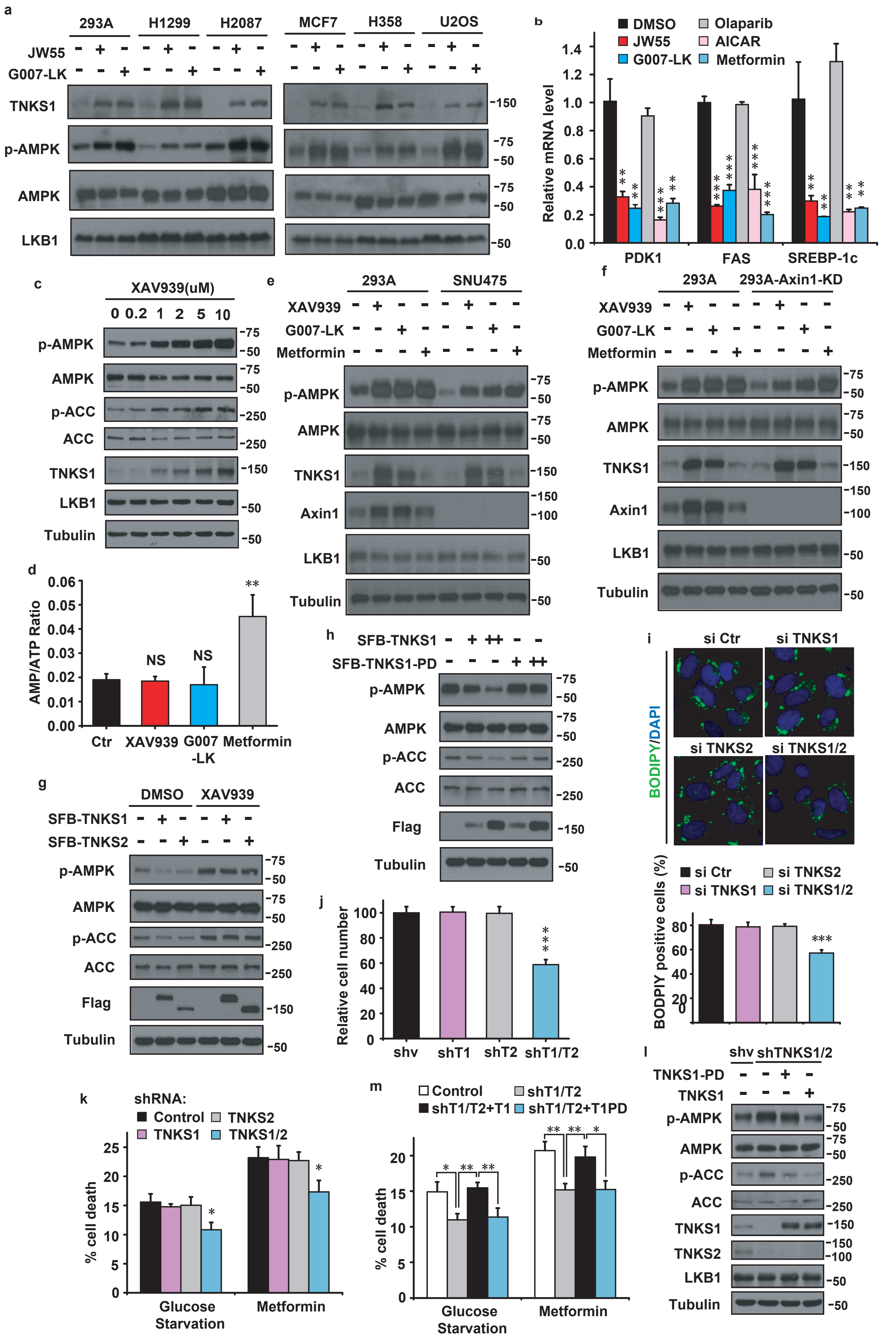
a
293A
H1299
H2087
MCF7
H358
U2OS
JW55
G007-LK
TNKS1
p-AMPK
AMPK
LKB1
-150
-75
-50
b
DMSO
JW55
G007-LK
Olaparib
AICAR
Metformin
Relative mRNA level
PDK1
FAS
SREBP-1c
c
XAV939(uM)
0 0.2 1 2 5 10
p-AMPK
AMPK
p-ACC
ACC
TNKS1
LKB1
Tubulin
-250
d
AMP/ATP Ratio
NS
**
Ctr
XAV939
G007-LK
Metformin
e
293A
SNU475
XAV939
G007-LK
Metformin
p-AMPK
AMPK
TNKS1
Axin1
LKB1
Tubulin
-100
f
293A
293A-Axin1-KD
g
DMSO
XAV939
SFB-TNKS1
SFB-TNKS2
Flag
h
SFB-TNKS1
SFB-TNKS1-PD
i
si Ctr
si TNKS1
si TNKS2
si TNKS1/2
BODIPY/DAPI
BODIPY positive cells (%)
***
j
Relative cell number
shv
shT1
shT2
shT1/T2
k
shRNA:
Control
TNKS1
TNKS2
TNKS1/2
% cell death
Glucose Starvation
Metformin
l
shv
shTNKS1/2
TNKS1-PD
TNKS1
TNKS2
m
Control
shT1/T2
shT1/T2+T1
shT1/T2+T1PD
% cell death
Glucose Starvation
Metformin

Supplementary Figure 1. Tankyrases Regulate AMPK Activation.

a, Tankyrase inhibitors induce AMPK activation in different cell lines. 293A/H1299/H2087/MCF7/H358/U2OScells were treated with tankyrase inhibitors JW55 and G007-LK and subjected to western blotting.

b, Tankyrase inhibitors suppress expression of AMPK negatively regulated downstream glycolytic genes. HEK293A cells were treated with indicated inhibitors. Relative expression of PDK1, FAS, and SREBP-1c were determined by qPCR.

c, XAV939 induces AMPK phosphorylation in a dose-dependent manner. HEK293A cells were treated with the indicated concentrations for 12 h, and the lysates was immunoblotted as indicated.

d, XAV939 and G007-LK don't affect cellular AMP/ATP ratio. HEK293A cells were treated with indicated inhibitors. Cellular level of AMP and ATP were determined by HPLC, and the AMP/ATP ratio was calculated.

e,f, Activation of AMPK pathway by tankyrase inhibitor does not mainly depend on Axin. The wild-type Axin–expressing cell line 293A and Axin-deficient cell line SNU475 (**e**) and 293A/293A-Axin-knockout variants (**f**) were treated with DMSO or XAV939 or G007-LK, or metformin and the indicated proteins were detected by western blotting.

g, TNKS1/2 inhibits phosphorylation of AMPK. Stable clones expressing SFB-tagged TNKS1 or TNKS2 were generated in HEK293T cells, which were treated with or without XAV939 and followed by western blotting.

h, Expression of TNKS1, but not TNKS1-PD, suppresses AMPK phosphorylation. HEK293T cells were transfected with increasing amounts of SFB-TNKS1 or TNKS1-PD and cell lysates were subjected to western blotting.

i, Double knockdown of TNKS1/2 inhibits lipid droplets formation. HEK293A cells were transduced with indicated siRNAs for 36 hrs and stained for lipid droplets (BODPIY 493/503, green) and nuclei (DAPI, blue). The results represent the means ±SD (n=4 independent

experiments).

j, Double -knockdown of TNKS1/2 suppresses proliferation of HEK293A cells. HEK293A cells were transduced with the indicated shRNAand the rate of cell growth was measured with an MTT assay; cell proliferation was determined relative to control cells.

k, Inhibition of energy stress–induced cell death by double depletion of TNKS1/2. HEK293A cells were transduced with the indicated shRNA, and then cells were seeded in 96-well plates and subjected to glucose starvation for 24 h or to 8 mM metformin for 3 days. Cell death was measured with an MTT assay and expressed as a percentage of the untreated controls.

l, Restore of TNKS1 but not TNKS1-PD mutant reversed TNKS1/2 KD induced AMPK activation. shRNA-resistant inducible TNKS1 or TNKS1-PD was transducted into TNKS1/2 knockdown HEK293A cells, cell lysates were subjected to western blotting.

m, Inhibition of energy stress induced cell death by depletion of TNKS1/2 is restored by expression of TNKS1, but not TNKS1-PD. shRNA-resistant inducible TNKS1 or TNKS1-PD was transducted into TNKS1/2 knockdown HEK293A cells, cells were seeded and subjected to glucose starvation for 24h, or to 8 mM Metformin for 3 days. Cell death was determined by MTT assay.

Statistical significance was determined by a two-tailed, unpaired Student's t-test. *P < 0.05; **P < 0.01; ***P < 0.001.

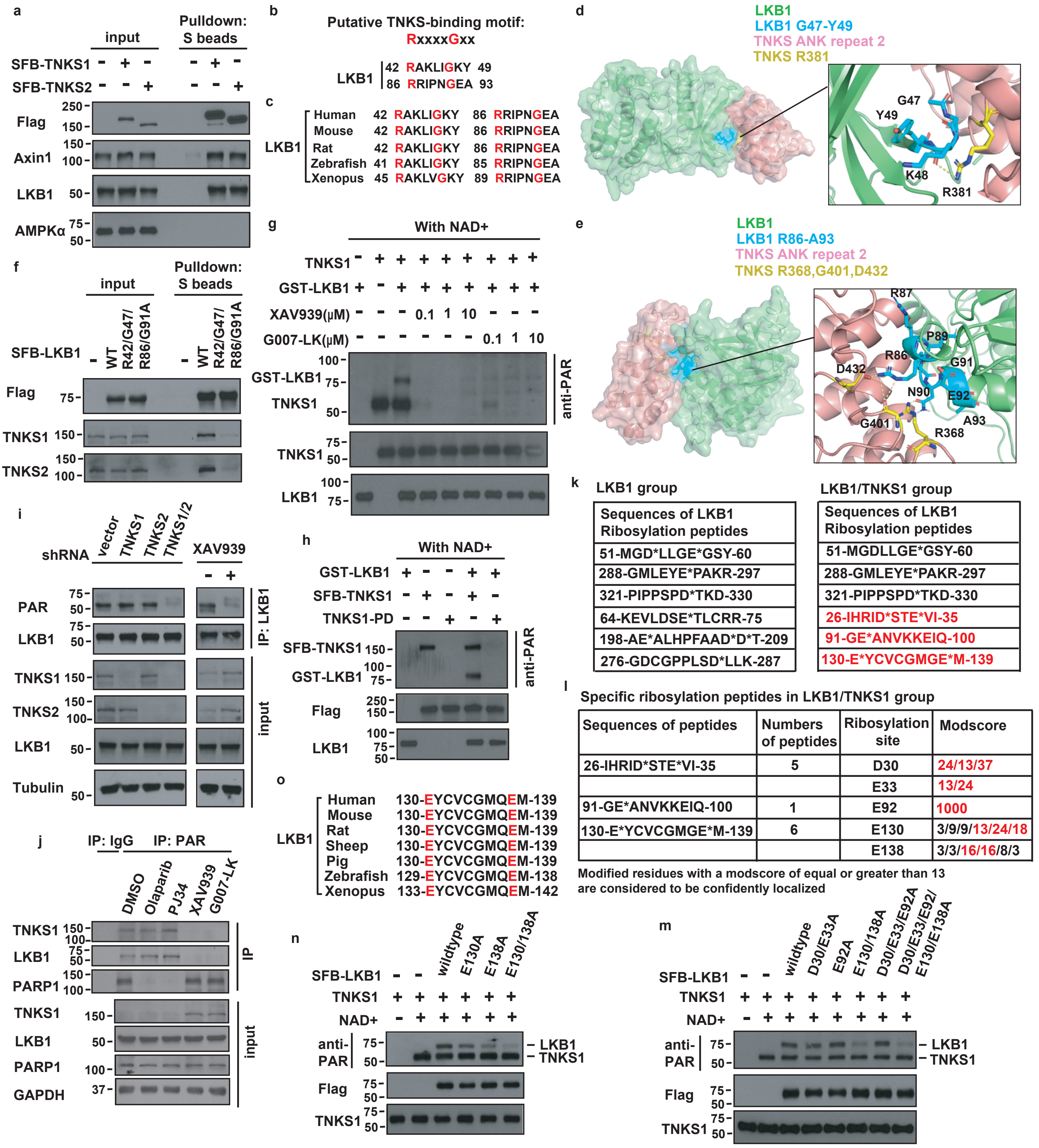

a
input
Pulldown: S beads
SFB-TNKS1
SFB-TNKS2
Flag
Axin1
LKB1
AMPKα
b
Putative TNKS-binding motif:
RxxxxGxx
LKB1 42 RAKLIGKY 49
86 RRIPNGEA 93
c
LKB1
Human 42 RAKLIGKY 86 RRIPNGEA
Mouse 42 RAKLIGKY 86 RRIPNGEA
Rat 42 RAKLIGKY 86 RRIPNGEA
Zebrafish 41 RAKLIGKY 85 RRIPNGEA
Xenopus 45 RAKLVGKY 89 RRIPNGEA
d
LKB1
LKB1 G47-Y49
TNKS ANK repeat 2
TNKS R381
G47
Y49
K48
R381
e
LKB1
LKB1 R86-A93
TNKS ANK repeat 2
TNKS R368,G401,D432
R87
P89
R86
G91
D432
N90
E92
A93
G401
R368
f
input
Pulldown: S beads
SFB-LKB1
WT
R42/G47/R86/G91A
Flag
TNKS1
TNKS2
g
With NAD+
TNKS1
GST-LKB1
XAV939(μM) 0.1 1 10
G007-LK(μM) 0.1 1 10
GST-LKB1
TNKS1
anti-PAR
TNKS1
LKB1
h
With NAD+
GST-LKB1
SFB-TNKS1
TNKS1-PD
SFB-TNKS1
GST-LKB1
anti-PAR
Flag
LKB1
i
shRNA
vector
TNKS1
TNKS2
TNKS1/2
XAV939
PAR
LKB1
IP: LKB1
TNKS1
TNKS2
LKB1
Tubulin
input
j
IP: IgG
IP: PAR
DMSO
Olaparib
PJ34
XAV939
G007-LK
TNKS1
LKB1
PARP1
IP
TNKS1
LKB1
PARP1
GAPDH
input
k
LKB1 group
Sequences of LKB1 Ribosylation peptides
51-MGD*LLGE*GSY-60
288-GMLEYE*PAKR-297
321-PIPPSPD*TKD-330
64-KEVLDSE*TLCRR-75
198-AE*ALHPFAAD*D*T-209
276-GDCGPPLSD*LLK-287
LKB1/TNKS1 group
Sequences of LKB1 Ribosylation peptides
51-MGDLLGE*GSY-60
288-GMLEYE*PAKR-297
321-PIPPSPD*TKD-330
26-IHRID*STE*VI-35
91-GE*ANVKKEIQ-100
130-E*YCVCGMGE*M-139
l
Specific ribosylation peptides in LKB1/TNKS1 group
Sequences of peptides | Numbers of peptides | Ribosylation site | Modscore
26-IHRID*STE*VI-35 | 5 | D30 | 24/13/37
E33 | 13/24
91-GE*ANVKKEIQ-100 | 1 | E92 | 1000
130-E*YCVCGMGE*M-139 | 6 | E130 | 3/9/9/13/24/18
E138 | 3/3/16/16/8/3
Modified residues with a modscore of equal or greater than 13 are considered to be confidently localized
o
LKB1
Human 130-EYCVCGMQEM-139
Mouse 130-EYCVCGMQEM-139
Rat 130-EYCVCGMQEM-139
Sheep 130-EYCVCGMQEM-139
Pig 130-EYCVCGMQEM-139
Zebrafish 129-EYCVCGMQEM-138
Xenopus 133-EYCVCGMQEM-142
n
SFB-LKB1
wildtype
E130A
E138A
E130/138A
TNKS1
NAD+
anti-PAR
LKB1
TNKS1
Flag
TNKS1
m
SFB-LKB1
wildtype
D30/E33A
E92A
E130/138A
D30/E33/E92A
D30/E33/E92/E130/E138A
TNKS1
NAD+
anti-PAR
LKB1
TNKS1
Flag
TNKS1

Supplementary Figure 2. LKB1 is a Substrate of Tankyrase.

a, LKB1 but not AMPKα interacts with TNKS1/2. 293T cells were transfected with SFB-tagged TNKS1 or TNKS2, and cell lysates were subjected to pulldown assays with the indicated antibodies.

b, The predicted tankyrase-binding motif for LKB1. Key residues in the putative tankyrase-binding motif are indicated in red.

c, The elements of the tankyrase-binding motif in LKB1 from different species. Key amino acids for each tankyrase-binding motif are indicated in red.

d,e, Structure of the LKB1/Tankyrase complex. The G47-Y49 (**d**) and R86-A92 (**e)** residues of LKB1 are located close to the interface between LKB1 and Tankyrase ANK repeats 2.

f, The tankyrase-binding motif of LKB1 is required for association between LKB1 and TNKS1/2. 293T cells were transfected with SFB-tagged LKB1 or tankyrase-binding motif point mutation of LKB1 (LKB1-R42/G47/R86/G91A), and cell lysates were subjected to pulldown assays.

g, TNKS1-induced ribosylation of LKB1 is blocked by tankyrase inhibitors XAV939 and G007-LK. In vitro ribosylation was assessed using E.coli purified GST-LKB1 and enzymatic active form of TNKS1(amino acids 1000-1328 with GST-tag, purchased from Sigma, catalog# SPR0422) with or without the indicated concentrations of XAV939/G007-LK, followed by western blotting.

h, The catalytic activity of TNKS1 is required for TNKS1-induced ribosylation of LKB1. Purified SFB-TNKS1, SFB-TNKS1-PD (full-length of TNKS1 or TNKS1-PD with SFB-tag, expressed and purified from 293T cells), and GST-LKB1 proteins were subjected to an in vitro ribosylation assay as indicated.

i, Depletion of TNKS1/2 or tankyrase inhibition diminishes LKB1 ribosylation in vivo. HEK293A cells were transduced with the indicated short hairpin RNAs (shRNA) or treated with or without XAV939, and cell lysates were subjected to immunoprecipitation/western blotting assays.

j, The ribosylation of TNKS1 and LKB1 were diminished by tankyrase inhibitors but not by PARP1/2 inhibitors. HEK293A cells were treated with indicated inhibitors, and cell lysates were

subjected to immunoprecipitation/western blotting assays.

k, LKB1 ribosylation peptides are listed from mass spectrometry results. Specific ribosylation peptides in LKB1/TNKS1 group are indicated in red.

l, Candidate ribosylation residues of LKB1 by TNKS1. A modscore of ≥13 is considered to confer more confidence in pinpointing the site of modification.

m,n, Map the key residues for TNKS1-induced LKB1 ribosylation. In vitro ribosylation was assessed with the indicated proteins and followed by western blotting.

o, Eements of the ribosylation sites in LKB1 from different species. The key amino acids are indicated in red.

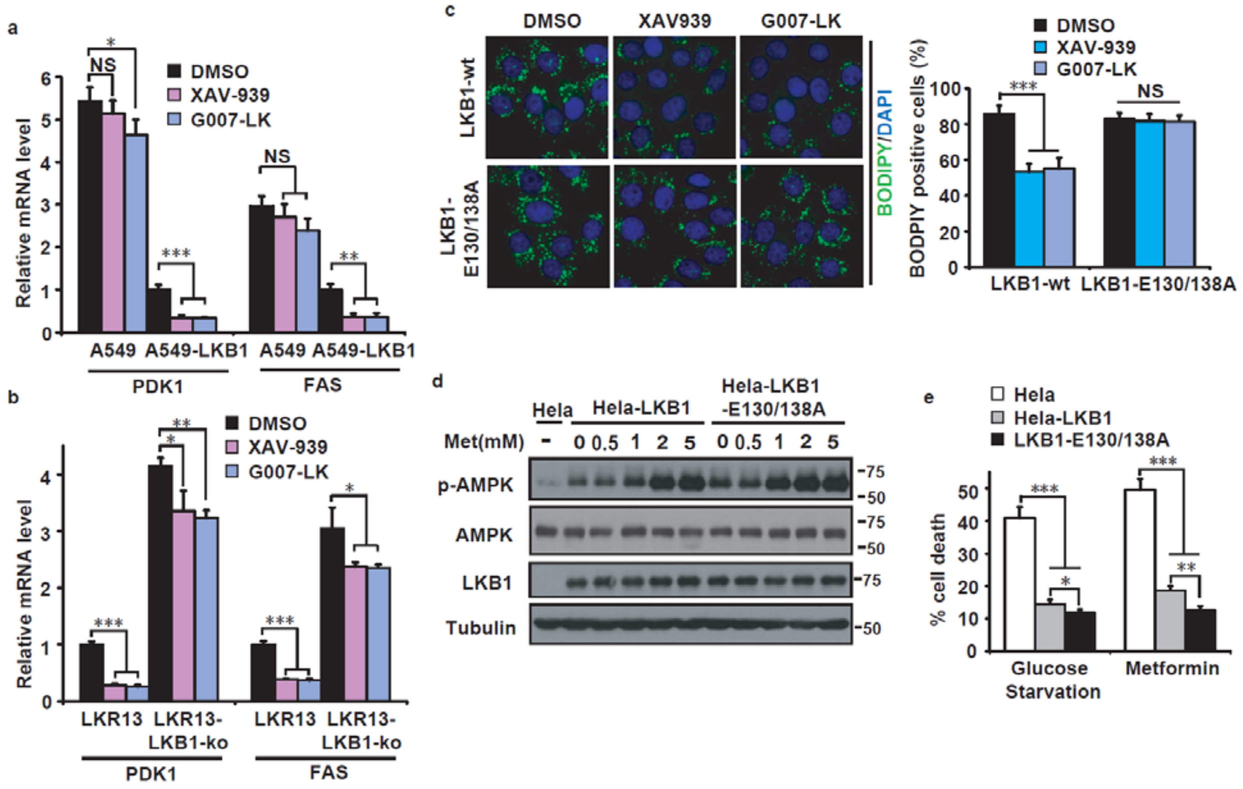

Supplementary Figure 3. LKB1 is Essential for Tankyrase-Mediated AMPK Regulation.

a, LKB1 is essential for the tankyrase inhibitor–mediated downregulation of AMPK target genes. A549 and A549-LKB1 cells were treated with the indicated inhibitors (5 μM) for 12 h, and relative expression levels of PDK1 and FAS were detected by qPCR.

b, LKB1 is required for tankyrase inhibitor–induced suppression of the expression of AMPK downstream genes in LKR13 cells. LKR13 and LKR13-LKB1-knockout cells were treated with the indicated inhibitors and relative expression levels of PDK1 and FAS were detected by qPCR. **c**, The LKB1-E130/138A mutant is resistant to tankyrase inhibitor–induced suppression of lipid droplet formation. HeLa cells stably expressing wild-type LKB1 or the LKB1-E130/138A mutant were treated with XAV939 or G007-LK and subjected to immunofluorescence staining for lipid droplets (BODPIY 493/503, green) and nuclei (DAPI, blue).

d, The LKB1-E130/138A mutant respond more strongly to metformin-induced AMPK phosphorylation. The indicated cells were treated or not treated with different concentrations of

metformin, and cell lysates were subjected to western blotting.

e, HeLa cells expressing the LKB1-E130/138A mutant were protected from energy stress–induced cell death. The indicated HeLa cells were subjected to glucose starvation or metformin treatment. Cell death was determined by MTT assay and expressed as a percentage of the untreated controls. Data are presented as means ±SD (n=3 independent experiments). Statistical significance was determined by a two-tailed, unpaired Student's t-test. *P < 0.05; **P < 0.01; ***P < 0.001.

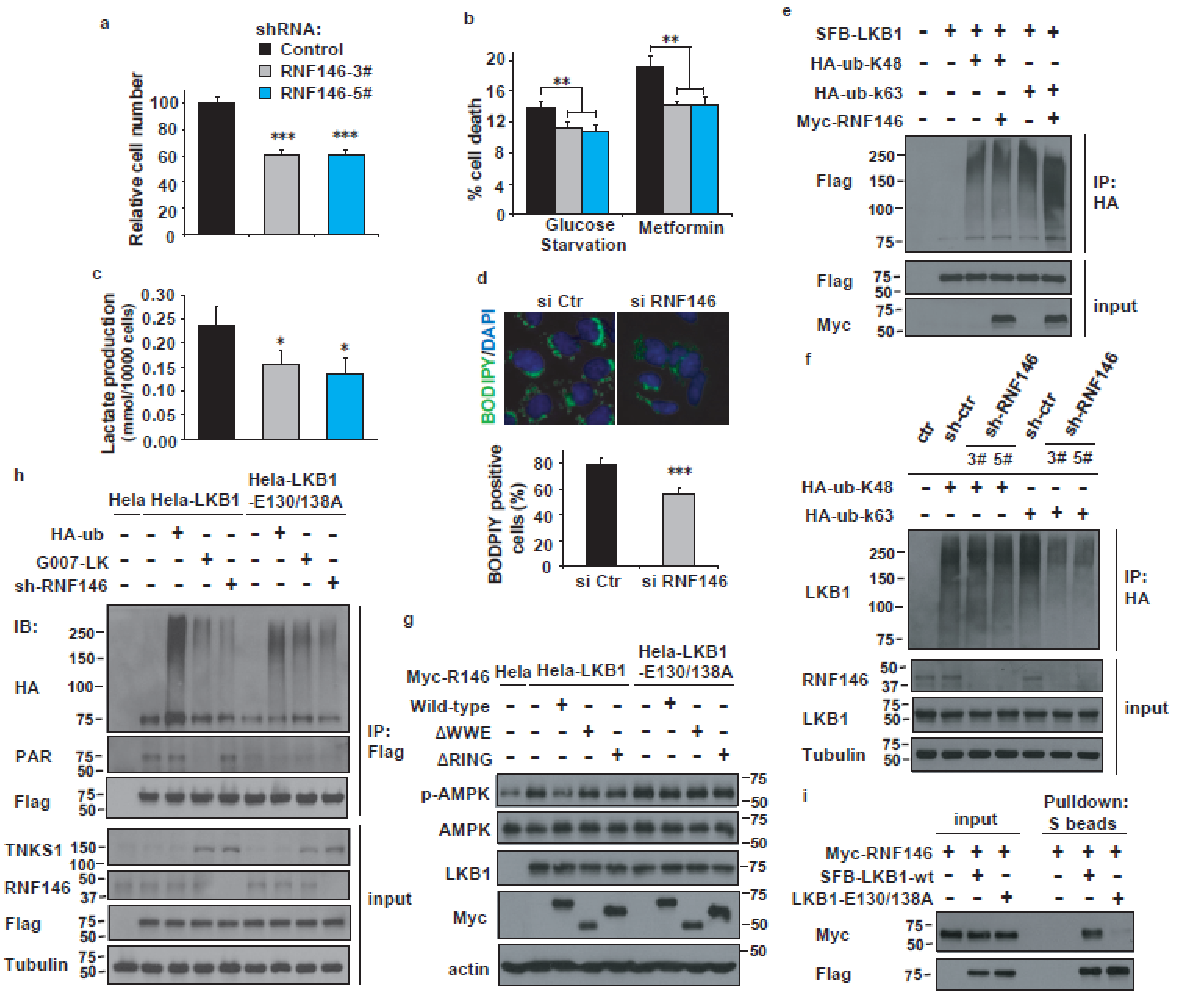

Supplementary Figure 4. RNF146 is Involved in Regulation of the LKB1-AMPK Pathway Through K63-Linked LKB1 Ubiquitination.

a, Depletion of RNF146 inhibits cell proliferation. HEK293A cells were transduced with indicated short hairpin RNAs (shRNA) and the rate of cell growth was detected using MTT assay, relative cell proliferation was determined relative to control cells. The results represent the means ±SD (n=3 independent experiments).

b, RNF146 depletion protects cells from energy stress–induced cell death. Control or RNF146-shRNA–infected HEK293A cells were subjected to glucose starvation or metformin treatment. Cell death was determined by MTT assay and expressed as a percentage of the untreated

controls. Data are presented as means ±SD (n=3 independent experiments).

c, Knockdown of RNF146 decreases lactate production. Lactate production was measured.

d, Depletion of RNF146 suppresses lipid droplet formation. HEK293A cells were transduced with the indicated siRNAs and stained for lipid droplets (BODPIY 493/503, green) and nuclei (DAPI, blue). The results represent the means ±SD (n=4 independent experiments).

e, RNF146 promotes LKB1 ubiquitination through K63-linkage. 293T cells were transfected with the indicated plasmids, cell lysates were subjected to an in vivo ubiquitination assay.

f, RNF146 depletion diminishes K63-linked LKB1 ubiquitinatio. HEK293A cells were co-transfected with the indicated shRNA and plasmids, cell lysates were subjected to an in vivo ubiquitination and followed by western blotting.

g, The LKB1-E130/138A mutant is resistant to RNF146-mediated suppression of the AMPK pathway. HeLa cells stably expressing wild-type LKB1 or the LKB1-E130/138A mutant were transfected with the indicated RNF146 constructs, and cell lysates were immunoblotted as indicated.

h, RNF146 knockdown or G007-LK treatment don't affect K63-linked ubiquitination of the LKB1-E130/138A mutant. 293T cells transfected with the shRNA-RNF146 or treated with G007-LK, cell lysates were subjected to an in vivo ubiquitination and followed by western blotting. **i**, RNF146 interacts with wild-type but not the E130/138A mutant of LKB1. Myc-tagged RNF146 was co-expressed with SFB-tagged wild-type or the E130/138A mutant of LKB1 in 293T cells, and cell lysates were subjected to pull-down assay.

Statistical significance was determined by a two-tailed, unpaired Student's t-test. *P < 0.05; **P < 0.01; ***P < 0.001.

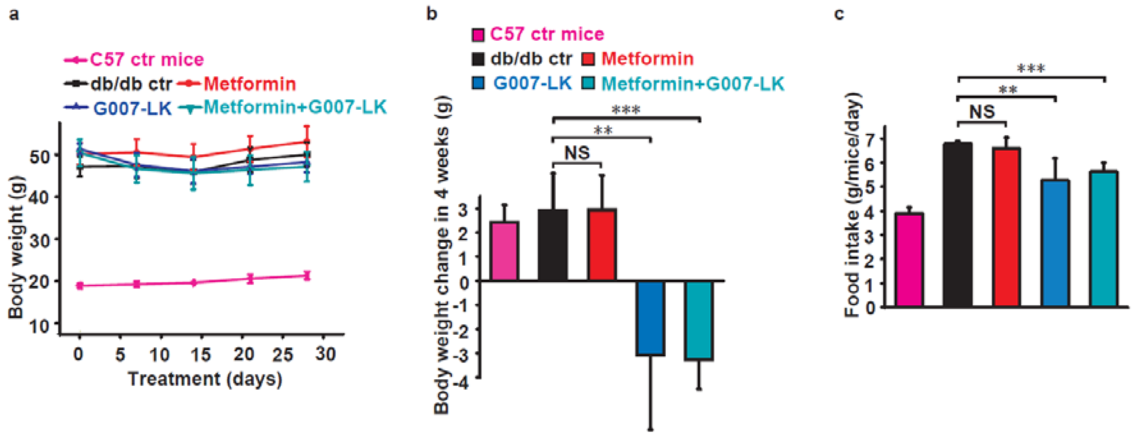

Supplementary Figure 5. G007-LK Affects Body Weight and Food Intake of db/db Mice.

a,b, Changes in body weight of db/db mice during treatment. The db/db mice were treated as indicated; body weights of each group of mice was measured at the indicated times (**a**) and changes in body weight throughout the treatment period were determined (**b**).

c, Daily food intake was measured after 3 weeks of treatment; data are presented as means ±SD (n=5 independent experiments).

Statistical significance was determined by a two-tailed, unpaired Student's t-test. *P < 0.05; **P < 0.01; ***P < 0.001.

a

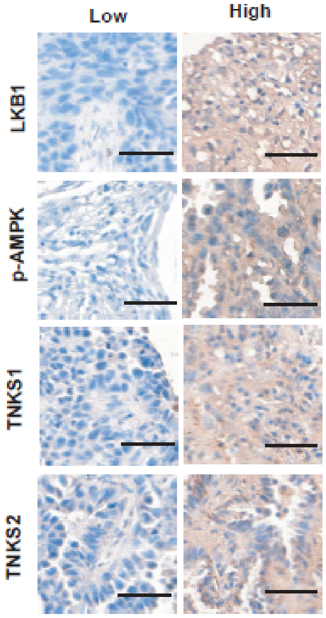

c

		LKB1 Low	LKB1 High	Total
p-AMPK	Low	84	35	119
	High	18	68	86
	Total	102	103	205

P < 1×10-5

		TNKS1 Low	TNKS1 High	Total
p-AMPK	Low	41	78	119
	High	39	47	86
	Total	80	125	205

P = 0.114

		TNKS2 Low	TNKS2 High	Total
p-AMPK	Low	34	85	119
	High	33	53	86
	Total	67	138	205

P = 0.139

b

	TNKS1 Low	TNKS1 High	Total
Normal lung	10	6 (37.5%)	16
Lung Carcinoma	70	119(62.9%)	189
Total	80	125	205

P = 0.044

	LKB1 Low	LKB1 High	Total
Normal lung	2	14 (87.5%)	16
Lung Carcinoma	100	89(47.1%)	189
Total	102	103	205

P = 0.001

	TNKS2 Low	TNKS2 High	Total
Normal lung	9	7 (43.7%)	16
Lung Carcinoma	58	131(69.3%)	189
Total	67	138	205

P = 0.036

	p-AMPK Low	p-AMPK High	Total
Normal lung	5	11 (68.7%)	16
Lung Carcinoma	114	75(39.6%)	189
Total	119	86	205

P = 0.023

Supplementary Figure 6. Overexpression of tankyrases in lung tumor tissues.

a, Immunohistochemical (IHC) staining of TNKS1/2, LKB1 and p-AMPK in normal lung tissues and lung carcinoma specimens as conducted in the US Biomax microarrays. Brown staining indicated positive immunoreactivity. Scale bars, 50 μM.

b, TNKS1/2, LKB1 and p-AMPK expression levels in normal lung tissues and lung carcinoma specimens.

c, Correlation between expression status of LKB1/p-AMPK, TNKS1/p-AMPK, and TNKS2/p-AMPK in human lung tumor samples.

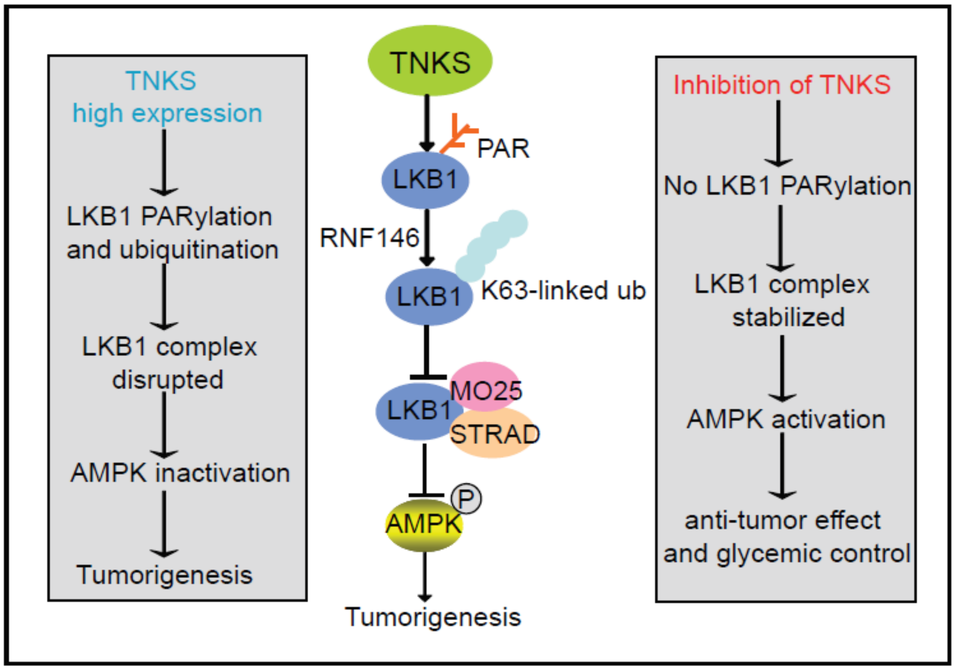

Supplementary Figure 7. Graphical working model for this work.
