# Supplementary material for: Tankyrase disrupts metabolic homeostasis and promotes tumorigenesis by inhibiting LKB1-AMPK signalling
Source: Nat Commun. 2019 Sep 25;10:4363. doi: 10.1038/s41467-019-12377-1 (PMC6761205; doi:10.1038/s41467-019-12377-1)
Supplement: Supplementary file 3 — Description of Additional Supplementary Files [file 41467_2019_12377_MOESM3_ESM.pdf]

Description of Additional Supplementary Files

File Name: Supplementary Data 1

Description: Drug Library Screened with a Phospho-AMPK Enzyme-Linked Immunosorbent Assay (Related to Figure 1a). Shown are the plate map of the drug screening library, the experimental set for the assay, and the results of the experiments are shown.

File Name: Supplementary Data 2

Description: Mass spectrum data of two groups (LKB1 alone as 2a, LKB1 with Tankyrase1 as 2b), and the comparison of two groups (2c) are shown (Related to Figure 1h).

File Name: Supplementary Data 3

Description: Statistical evaluation of relative ratios of western blot in Fig 4. Shown are the statistical evaluation of western blotting bands densitometry relative ratios in Fig 4. Data were analyzed by the One-way ANOVA test and we used an F-test to compare variances, and a P value of ＜0.05 was considered to indicate statistically significant differences.

File Name: Supplementary Data 4

Description: The clinical and pathological features of tissue specimens in Figure 6g.h are listed in Supplementary Data 4a, b. 4a, Lung adenocarcinoma (HLugA180Su05). 4b, Lung squamous cell carcinoma (HLug-Squ150Sur-02).
