# Supplementary material for: Tankyrase disrupts metabolic homeostasis and promotes tumorigenesis by inhibiting LKB1-AMPK signalling
Source: Nat Commun. 2019 Sep 25;10:4363. doi: 10.1038/s41467-019-12377-1 (PMC6761205; doi:10.1038/s41467-019-12377-1)
Supplement: Supplementary file 8 — Reporting Summary [file 41467_2019_12377_MOESM8_ESM.pdf]

nature research

Corresponding author(s): Nan Li, Junjie Chen, Steven H Lin

Reporting Summary

Nature Research wishes to improve the reproducibility of the work that we publish. This form provides structure for consistency and transparency in reporting. For further information on Nature Research policies, see Authors & Referees and the Editorial Policy Checklist.

Statistical parameters

When statistical analyses are reported, confirm that the following items are present in the relevant location (e.g. figure legend, table legend, main text, or Methods section).

n/a	Confirmed	
☐	☒	The exact sample size (*n*) for each experimental group/condition, given as a discrete number and unit of measurement
☐	☒	An indication of whether measurements were taken from distinct samples or whether the same sample was measured repeatedly
☐	☒	The statistical test(s) used AND whether they are one- or two-sided<br>*Only common tests should be described solely by name; describe more complex techniques in the Methods section.*
☐	☒	A description of all covariates tested
☐	☒	A description of any assumptions or corrections, such as tests of normality and adjustment for multiple comparisons
☐	☒	A full description of the statistics including central tendency (e.g. means) or other basic estimates (e.g. regression coefficient) AND variation (e.g. standard deviation) or associated estimates of uncertainty (e.g. confidence intervals)
☐	☒	For null hypothesis testing, the test statistic (e.g. *F*, *t*, *r*) with confidence intervals, effect sizes, degrees of freedom and *P* value noted<br>*Give P values as exact values whenever suitable.*
☒	☐	For Bayesian analysis, information on the choice of priors and Markov chain Monte Carlo settings
☒	☐	For hierarchical and complex designs, identification of the appropriate level for tests and full reporting of outcomes
☐	☒	Estimates of effect sizes (e.g. Cohen's *d*, Pearson's *r*), indicating how they were calculated
☐	☒	Clearly defined error bars<br>*State explicitly what error bars represent (e.g. SD, SE, CI)*

Our web collection on statistics for biologists may be useful.

Software and code

Policy information about availability of computer code

Data collection	No commercial, open source and custom code used to collect the data.
Data analysis	n/a

For manuscripts utilizing custom algorithms or software that are central to the research but not yet described in published literature, software must be made available to editors/reviewers upon request. We strongly encourage code deposition in a community repository (e.g. GitHub). See the Nature Research guidelines for submitting code & software for further information.

Data

Policy information about availability of data

All manuscripts must include a data availability statement. This statement should provide the following information, where applicable:

- Accession codes, unique identifiers, or web links for publicly available datasets
- A list of figures that have associated raw data
- A description of any restrictions on data availability

All relevant data not presented in the main figures or Supplementary Data are available from the corresponding author on reasonable request.

Field-specific reporting

Please select the best fit for your research. If you are not sure, read the appropriate sections before making your selection.

☒ Life sciences ☐ Behavioural & social sciences ☐ Ecological, evolutionary & environmental sciences

For a reference copy of the document with all sections, see nature.com/authors/policies/ReportingSummary-flat.pdf

Life sciences study design

All studies must disclose on these points even when the disclosure is negative.

Sample size	Sample size were determined with statistic analysis.
Data exclusions	No data were excluded from the analysis.
Replication	All experiments were repeated by at least three times. The quantification results were evaluated with statistic analysis among repeats.
Randomization	We did not exclude any samples or animals from the analysis, and we used random assignment of samples or animals to different groups.
Blinding	Quantification of the xenograft tumor size was done by a person who was blinded to genotype and treatment of the samples.

Reporting for specific materials, systems and methods

Materials & experimental systems

n/a	Involved in the study	
☒	☐	Unique biological materials
☐	☒	Antibodies
☐	☒	Eukaryotic cell lines
☒	☐	Palaeontology
☐	☒	Animals and other organisms
☒	☐	Human research participants

Methods

n/a	Involved in the study	
☒	☐	ChIP-seq
☒	☐	Flow cytometry
☒	☐	MRI-based neuroimaging

Antibodies

Antibodies used	For western blotting, rabbit anti-TNKS2 antibody was raised by immunizing rabbits with GST-TNKS2 fusion protein containing amino acids 527-776 of human TNKS2. Anti-RNF146 (ab201212, 1:500 dilution) was obtained from abcam. Anti-HA (H9658, 1:2000 dilution), anti-α-tubulin (T6199, 1:3000 dilution), anti-Flag (M2) (F3165, 1:5000 dilution) and anti-β-actin (A2228, 1:2000 dilution) were purchased from Sigma-Aldrich. Anti-LKB1 (3047S, 1:1000 dilution), anti-phospho-LKB1 (T189) (3054S, 1:1000 dilution), anti-phospho-LKB1 (S334) (3055S, 1:1000 dilution), anti-phospho-LKB1 (S428) (3482S, 1:1000 dilution), anti-Axin1 (2074S, 1:1000 dilution), anti-AMPKα (2532S, 1:1000 dilution), anti-phospho-AMPKα (T172) (2535S, 1:1000 dilution), anti-ACC (3662S, 1:1000 dilution), anti-phospho-ACC (S79) (3661S, 1:1000 dilution), and anti-MO25α (2716S, 1:3000 dilution) were obtained from Cell Signaling Technology. Anti-Myc (sc-40, 1:1000 dilution), anti-LKB1 (sc-374300, 1:1000 dilution), anti-TNKS (sc-8337, 1:1000 dilution), and anti-GST (sc-138, 1:1000 dilution) were purchased from Santa Cruz Biotechnology. Anti-ubiquitin-K63 (05-1313, 1:500 dilution) was obtained from Millipore; anti-STRADα (PA572175, 1:500 dilution) was obtained from Thermo Fisher; and anti-PAR (4335-MC-100, 1:1000 dilution) was obtained from Trevigen.
Validation	Each primary antibody for the species and application has the validation statement on the manufacturer's website and conformed by relevant citations.

Eukaryotic cell lines

Policy information about cell lines

Cell line source(s)	The HEK293T, HEK293A, H1299, H2087, MCF7, H358, U2OS, SNU475, HeLa, and A549 cell lines were purchased from the American Type Culture Collection (ATCC). The LKR13 mouse lung cancer cells were kindly provided by Dr. Tyler Jacks (Massachusetts Institute of Technology) .
Authentication	In the manuscript, we described that "The LKR13 mouse lung cancer cells were kindly provided by Dr. Tyler Jacks

Authentication	(Massachusetts Institute of Technology)". All the other cell lines were ordered from ATCC with authentication or generated by our lab.
Mycoplasma contamination	Confirmed
Commonly misidentified lines (See ICLAC register)	None

Animals and other organisms

Policy information about studies involving animals; ARRIVE guidelines recommended for reporting animal research

Laboratory animals	6- to 8-wk-old female BALB/c nude mice, ten-week-old male C57BL/KsJ-Leprdb/Leprdb (db/db), male 129S background mice were purchased from the Jackson Laboratory.
Wild animals	*Provide details on animals observed in or captured in the field; report species, sex and age where possible. Describe how animals were caught and transported and what happened to captive animals after the study (if killed, explain why and describe method; if released, say where and when) OR state that the study did not involve wild animals.*
Field-collected samples	*For laboratory work with field-collected samples, describe all relevant parameters such as housing, maintenance, temperature, photoperiod and end-of-experiment protocol OR state that the study did not involve samples collected from the field.*
